# The relationship between circulating concentrations of interleukin 17 and C reactive protein in chronic spontaneous urticaria

**DOI:** 10.1186/s13223-017-0197-6

**Published:** 2017-05-10

**Authors:** A. Grzanka, A. Damasiewicz-Bodzek, A. Kasperska-Zajac

**Affiliations:** 1 Department of Internal Diseases, Dermatology and Allergology, School of Medicine with Division of Dentistry in Zabrze, Medical University of Silesia in Katowice, Katowice, Poland; 2 Department of Chemistry, School of Medicine with Division of Dentistry in Zabrze, Medical University of Silesia in Katowice, Katowice, Poland

**Keywords:** Chronic spontaneous urticaria, C-reactive protein, IL-17 interleukin

## Abstract

**Background:**

Up-regulation of interleukin 17 (IL-17) family cytokines and acute phase response have been observed in patients with chronic spontaneous urticaria (CSU). It has been demonstrated that IL-17 stimulates C-reactive protein (CRP) expression.

**Aim:**

To determine relationship between circulating concentrations of IL-17 and CRP in CSU.

**Methods:**

Concentrations of IL-17 in plasma and CRP in serum were measured in patients with CSU of varying severity and in the healthy subjects.

**Results:**

IL-17 and CRP concentrations were significantly higher in CSU patients as compared to the healthy subjects. In addition, there were significant differences in IL-17 and CRP concentrations between CSU patients with mild, moderate-severe symptoms and the healthy subjects. CRP did not correlate significantly with IL-17.

**Conclusions:**

Increased circulating IL-17 concentration may represent an independent index of systemic inflammatory response in CSU, which is not related to increased CRP concentration.

## Background

Chronic spontaneous urticaria (CSU) is a complex, systemic disease with a multifactorial etiopathogenesis associated with autoimmune and inflammatory phenomena [1–3].

It has been hypothesized that CSU is associated with a T helper cell 17 (Th17)—mediated immune response [4, 5], which is characterized by production of interleukin 17 (IL-17). IL-17 is linked to pathogenesis of inflammatory/autoimmune diseases [6, 7].

The pro-inflammatory cytokine plays an important role in synthesis of many mediators, including IL-6, IL-8, IL-1β and TNF-α [6, 8], which may reflect the inflammatory state in CSU [2, 4, 5].

Serum C reactive protein (CRP)—the best marker of acute phase response and IL-17 concentrations, may be associated with CSU activity [2, 5]. It has been suggested that IL-17-CRP signaling may play a role in chronic inflammatory conditions [9], although, its regulation and function in CSU have remained unclear. Therefore, the aim of this study was to determine the relationship between circulating concentrations of IL-17 and CRP in CSU patients.

## Methods

52 CSU patients (14 men and 38 women; median age: 38 years, range: 24–50) with a median disease duration of 2.5 years were enrolled in the study.

The patients underwent the following tests and procedures: routine laboratory tests, stool (for parasites and *H. pylori*), antinuclear and antithyroid microsomal antibodies, thyroid function, chest X-ray and abdominal ultrasonography, hepatitis serology, autologous serum skin test (ASST) [10]. Additionally, dental, gynecological and ENT consultations were performed.

Urticaria activity score according to EAACI/GALEN/EDF guidelines was estimated during four days and on the blood sampling day and graded as follows: mild (0–8), moderate (9–16) and severe (17–24). The study comprised 28 patients with mild CSU and 24 patients with moderate-severe symptoms.

H1-antihistamine drugs were withdrawn at least 4 days before blood sampling. None of the patients had been taking immunosuppressants or any other drugs, for at least 8 weeks before the study.

The control group comprised 21 sex-, age- and BMI (<30) matched the healthy subjects.

The Ethics Committee of the Medical University of Silesia approved of the study and written, informed consent was obtained from all the subjects participating.

### Blood collection

Blood samples were taken on fasting, from elbow veins using tubes with anticoagulant. Plasma obtained by centrifugation were stored at −85 °C until the tests were performed.

### Assay of interleukin-17 (IL-17)

Concentrations of IL-17 in plasma samples were measured by ELISA method using commercially available kits (Quantikine Human CXCL8/IL-8 ELISA kit from R&D Systems, MN, USA) according to manufacturers’ detailed instructions. The variance coefficients for intra-assay and inter-assay were below 8 and 10% respectively. Sensitivity of the kit is 15 pg/ml.

### CRP assay

Serum C-reactive protein (CRP) concentrations were measured using Roche/Hitachi cobas c system. Normal lab ranges: lower than 5.0 mg/l.

### Statistical analysis

The obtained results were presented with the use of basic parameters of descriptive statistics. Normal distribution of data was measured using Shapiro–Wilk’s test. Independent data between two groups of patients with CSU and the controls were compared using non-parametric U Mann–Whitney test. Independent data between three groups of patients with mildCSU, moderate-severe CSU and the controls were compared using non-parametric ANOVA rang Kruskal–Wallis’s test. The Spearman’s rank test was used for correlations. The p < 0.05 was considered statistically significant. Calculations were performed with STATISTICA for Windows 10.0 software (StatSoft, Cracow, Poland).

## Results

### Plasma IL-17 concentration

**Fig. 1:**
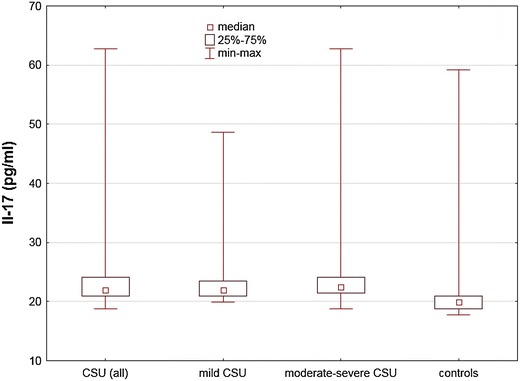
Plasma IL-17 concentration in chronic spontaneous urticaria (CSU) patients with different disease activity and in the healthy subjects. CSU vs. controls, p < 0.001; moderate-severe CSU patients and mild CSU vs. controls, p < 0.005; mild CSU vs. moderate-severe CSU, p > 0.05

Plasma concentrations of IL-17 were significantly higher in CSU patients as compared with the healthy subjects [median and quartile range/min–max: 21.97 (20.92–24.98/18.85–62.73) vs. 19.88 (18.85–20.92/17.82–59.16) pg/ml, p < 0.001; Fig. 1].

IL-17 plasma concentrations were significantly higher in moderate-severe and mild CSU patients as compared with the healthy subjects [median and quartile range/min–max: 22.49 (20.92–24.08/18.85–62.73) and 21.97 (20.92–23.55/19.28–48.60) vs. 19.88 (18.85–20.92/17.82–59.16) pg/ml, p < 0.005]. Concentrations of IL-17 in mild CSU patients did not differ significantly versus moderate-severe CSU patients [median and quartile range/min–max: 21.97 (20.92–23.55/19.28–48.60) vs. 22.49 (20.92–24.08/18.85–62.73) pg/ml, p > 0.05; Fig. 1].

No significant differences in IL-17 concentrations between ASST(+) and ASST(−) CSU patients (selected according to the similar UAS) were observed.

### Serum CRP concentration

**Fig. 2:**
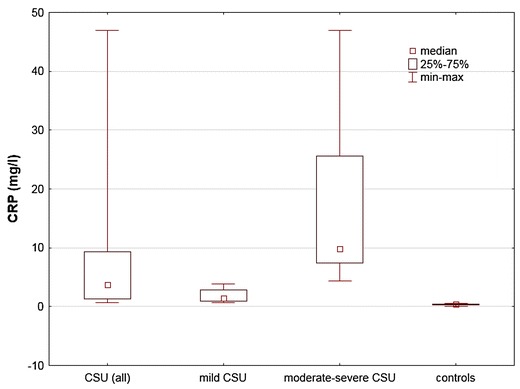
CRP concentration in serum of chronic spontaneous urticaria (CSU) patients and in the healthy subjects. CSU vs. controls, p < 0.001; mild CSU vs. moderate-severe CSU vs. controls, p < 0.001

Serum CRP concentrations were significantly higher in CSU patients as compared with the healthy subjects [median and quartile range/min–max: 3.8 (1.30–9.20/0.70–46.90) vs. 0.4 (0.20–0.40/0.10–0.60) mg/l, p < 0.001]. In addition, there were significant differences in serum CRP concentration between CSU patients with mild, moderate-severe symptoms and the healthy subjects [median and quartile range/min–max: 1.4 (20.92–23.55/19.28–48.60) vs. 9.8 (20.92–24.08/18.85–62.73) vs. 0.4 (0.20–0.40/0.10–0.60) mg/l, respectively; p < 0.001; Fig. 2].

### Correlation between values of CRP and IL-17 in patients with CSU

CRP did not correlate significantly with IL-17 (r = 0.12, p = 0.41).

## Discussion

In our study, plasma IL-17 concentrations were significantly higher in CSU patients as compared with the healthy subjects. In addition, there were significant differences in IL-17 concentrations between CSU patients with mild, moderate-severe symptoms and the healthy subjects. However, IL-17 values did not show significant differences between the groups of mild and moderate-severe disease. Atwa et al. reported increased IL-17 serum concentrations in CSU patients that paralleled disease severity and ASST response [5]. In addition, there is some convincing evidence to prove that acute phase activation is associated with CSU activity/severity and may be involved in pathogenesis of the disease [2]. Based on association between acute phase response and IL-17, we addressed the question whether increased IL-17 and CRP are related to each other.

It has been demonstrated that IL-17 can stimulate CRP and IL-6 expression, suggesting that the cross-talk between IL-17 and CRP/IL-6 may further amplify an inflammatory cascade [9, 11].

It is known that IL-6-CRP signaling plays an important role in CSU. Interestingly, it has been suggested that IL-17 stimulates CRP expression independently of IL-1 and IL-6. However, IL-17 may act in synergy with IL-6, which potentiates IL-17-mediated CRP expression. [9].

The link between IL-6 and CRP has been confirmed in CSU [2]. In the present study, we did not observe any association between CRP and IL-17 concentrations in CSU patients, contrary to the expectations.

The lack of correlation between IL-17 and CRP suggests, that increased IL-17 concentration cannot be regarded as a simple reflection of acute phase activation. IL-17 seems to be related to a wider spectrum of immune/inflammatory responses and provides independent information from the acute phase response in CSU. CRP concentration is increased in CSU patients and it may reflect the disease activity/severity. However, mild urticarial processes can result in only slight elevation (within normal range) of the acute phase response marker.

Th17 cells and their signature cytokine—IL-17 play a key role in initiation and maintenance of autoimmune and inflammatory processes in different diseases, leading to secretion of several inflammatory factors and autoantibody production [7, 12].

The source of IL-17 in CSU is unclear. Elevated IL-17 is likely to result from activation of different cells involved in urticarial processes, especially mast cells [13]. It has been demonstrated that mast cells primarily MC_TC_ cells, are the most numerous cells containing IL-17 in human skin [13]. These cells are able to produce IL-17 upon stimulation with various stimuli, including TNF-alpha and C5a [13]. IL-17 is produced by many other cell types including lymphocytes, neutrophils [14, 15].

All of these factors are upregulated and may play role in pathogenesis of immune-inflammatory response in CSU [1, 16].

The current study confirms that circulating IL-17 concentrations are increased in CSU patients [4, 5]. Similar observations have been made in various inflammatory and autoimmune diseases providing the rationale for development of the anti-IL-17A monoclonal antibody [6, 14]. Therefore, it seems that biological agents targeting IL-17A signalling pathways may represent a potential approach for treating CSU.

## Conclusions

The current finding confirms that circulating IL-17 concentration is increased in CSU patients and may represent an independent index of systemic inflammatory response, which is not related to increased CRP concentration.

## Abbreviations

CSU: chronic spontaneous urticaria
CRP: C reactive protein
IL-17: interleukin 17
UAS: urticaria activity score
ASST: autologous serum skin test
